# Obesity in chronic spinal cord injury is associated with poorer body composition and increased risk of cardiometabolic disease

**DOI:** 10.1038/s41598-025-13593-0

**Published:** 2025-08-08

**Authors:** Nicholas Dietz, Maxwell Boakye, Martin F. Bjurström, Beatrice Ugiliweneza, Shirish Barve, Sriprakash Mokshagundam

**Affiliations:** 1 Department of Neurosurgery, University of Louisville, Louisville, KY USA; 2 Department of Surgical Sciences, Clinical Pain Research, Uppsala University, Uppsala, Sweden; 3 Department of Medicine, University of Louisville, Louisville, KY USA; 4 Department of Endocrinology, University of Louisville, Louisville, KY USA

**Keywords:** Traumatic spinal cord injury, Metabolism, Cardiometabolic disease, Neuroscience, Physiology

## Abstract

With increased longevity after spinal cord injury (SCI), cardiovascular disease has emerged as a major cause of morbidity and mortality. We evaluate the association of body composition and injury level with cardiometabolic disease (CMD) risk factors. Sixty-two individuals (69% male, 31% female) with chronic SCI (mean duration 7.4, SD *±* 5.8 years) were recruited. Mean age of participants was 34.4 *±* 12.1 years with BMI of 23.8 *±* 5, while 64% had BMI < 25 and 11% >30. Total and percent truncal fat correlated positively (*p* < 0.05) with serum triglycerides, non-high-density lipid cholesterol, c-reactive protein (CRP), oral glucose tolerance test (OGTT), and measures of insulin resistance. Those with obesity in SCI (defined as BMI *≥* 22) had increased total and trunk mass and fat percentage, unfavorable lipid profiles and evidence of insulin insensitivity. Total fat was associated with CMD risk factors, including insulin resistance (OGTT 60 min *r* = 0.47, *p* < 0.05; homeostasis model assessment [HOMA] *r* = 0.62, *p* < 0.05), serum triglycerides (*r* = 0.31, *p* < 0.05), and inflammation (CRP *r* = 0.43, *p* < 0.05). Obesity in SCI related to higher CMD risk, while time since injury and injury level (paraplegia versus tetraplegia) did not. Future studies may evaluate roles of nutrition, exercise, sleep-promotion, and pharmaceuticals to lower neurogenic obesity and chronic CMD risk.

**Supplementary Information:**

The online version contains supplementary material available at 10.1038/s41598-025-13593-0.

## Introduction

Sequelae of spinal cord injury (SCI) include dramatic changes in body composition and metabolic dysfunction that lead to higher risk of cardiovascular disease and decreased life expectancy^1–4^. Within weeks of injury, decreases in muscle cross sectional area and free fat mass as well as increased fat infiltration contribute to neurogenic obesity^5–9^. A positive energy balance with reduced basal metabolic rate and resting energy expenditure ultimately result in higher visceral adipose tissue^5,10^. Additionally, the loss of muscle mass coupled with the accumulation of visceral fat are believed to drive a metabolically unhealthy obesity with insulin resistance and unfavorable lipid profiles^5,11,12^. This trend appears to correspond with level of injury such that individuals with tetraplegia have higher percentages of central and visceral adiposity as well as lipid dysfunction compared with those with paraplegia^3,13–15^. In those with SCI, body mass index (BMI) > 22.0 is considered to define obesity that accounts for the metabolic consequences of increased fat mass percentage and dramatic skeletal muscle atrophy^16^.

Truncal fat and central obesity have been linked to pro-inflammatory cytokines and increased risk of cardiometabolic disease (CMD) in the general population^17^. While central or visceral obesity may represent primary sources of inflammation, it is unclear what other factors may play a role in driving or sustaining the inflammatory state in SCI^8,18^. Further, CMD, a key outcome of body composition dysregulation, is a leading cause of death in SCI and is observed in 72% of those with chronic SCI compared to 34% of able-bodied adults^2–5,12,19^. Individuals with SCI experience CMD from insulin insensitivity, dyslipidemia, and vascular disease earlier and with greater incidence and severity^1,12,13^. Inflammatory changes underlie the difference between metabolically unhealthy versus metabolically healthy obese in the general population^20^. Specifically, adipokines signal regional pro-inflammatory changes, including macrophage inflammatory protein-1 alpha (MIP-1 α), monocyte chemoattractant protein-1 (MCP-1), interleukin 8 (IL-8), and tumor necrosis factor alpha (TNF-α) with infiltration into adipose tissue that dysregulate systemic metabolism and lead to insulin resistance and vascular dysfunction^8,20,21^. Similarly, levels of pro-inflammatory c-reactive protein (CRP) and IL-6 are associated with chronic SCI and contribute to the milieu of chronic inflammation, lipid metabolism dysregulation, and vascular injury^22,23^. Ectopic fat stores contribute to systemic inflammation and metabolic syndrome^24^. Further, gut microbiome changes accompany SCI and lead to bowel dysfunction and resulting gut dysbiosis characterized by decreases in butyrate-producing “healthy gut bacteria”^25,26^. However, few studies have investigated the relationship between body profile and immune markers or changes to the microbiome, as well as the role certain systemic inflammatory markers may have in duration of SCI.

In the present study, 62 persons with chronic spinal cord injury were recruited and analyzed to assess body composition, level of injury, metabolic profile, inflammation and risk of CMD. Specifically, relationships between body composition, serum inflammatory markers, bowel dysfunction and transit, stratified by BMI and relevant injury severity across individuals with paraplegia and tetraplegia are investigated. The aim of the description study was to assess correlations between inflammatory markers and body composition changes and determine the extent to which level of injury, time since injury, or body composition may influence these changes in inflammation. We hypothesize that greater BMI will relate to higher systemic inflammatory changes and that bowel dysfunction will relate to worse inflammatory changes and body pod metrics.

## Methods

### Study design

Cross-sectional cohort study design.

### Patient selection

Adult patients > 18 years old with chronic traumatic spinal cord injury (injury duration > 2 years) American Spinal Injury Association (ASIA) grades A-C were included in the study. ASIA A is complete motor and sensory loss below the level of the lesion while ASIA B refers to motor loss but sensation present below the level of the lesion. ASIA C relates to sensation below the level of the lesion with motor strength less than 3/5 in half of key muscle groups below level of the lesion. Exclusion criteria included SCI from non-traumatic etiology (i.e. neoplasm, infection, etc.) or active infection that may confound inflammatory markers. The institutional review board (IRB) approved the present study (IRB# 16.0179). All participants were recruited by phone and medically approved for enrollment in a prospective outpatient, standardized program from 2009 to 2020, as previously described^27^. Patients with chronic spinal cord injury were recruited to participate in a spinal cord epidural stimulator trial with a rehabilitation regimen 2–3 weeks after implantation. Data was taken from this recruitment study in a retrospective fashion, and the need to obtain additional informed consent was waived by the University of Louisville IRB. All methods were performed in accordance with the relevant guidelines and regulations of the institution and journal.

### Metabolic measures

Body composition was measured by dual energy X-ray absorptiometry (DEXA) scan. Metabolic profile, lipids, high-sensitivity CRP (hsCRP), a battery of inflammatory cytokines were assessed through analysis of serum samples. American Diabetes Association criteria for normal, impaired fasting glucose (IFG), impaired glucose tolerance (IGT), and diabetes mellitus (DM) were used^28^. Oral Glucose Tolerance Test (OGTT) was performed with 75 gram glucose load and blood samples drawn at 0, 30, 60, 90, and 120 min for measurement of glucose and insulin (Table 1). Insulin Resistance was estimated using the following: Homeostasis Model Assessment 2 (HOMA2); Homeostasis Model Assessment of Insulin Resistance (HOMA-IR); Matsuda Index. Matsuda Index is a measure of peripheral or whole-body insulin sensitivity that is calculated by non-fasting glucose tolerance test. Values greater than 4.0 indicate increased insulin insensitivity^29^. Insulin Secretion was estimated using Insulinogenic Index and Disposition Index. Diet and calorie breakdown including macronutrients were recorded for groups, as was the neurogenic bowel score.

**Table 1:** Metabolic measures with reference range and meaning.

Measure	Recommended normal value threshold	Meaning
HOMA2	> 2.0	Estimates hepatic influence of insulin resistance for which values greater than 2.0 indicate insulin resistance
HOMA-IR	0.5–2.5	Estimates insulin resistance where values greater than 2.5 correlate with greater severity of insulin resistance
Matsuda	> 4.0	Peripheral or whole-body insulin calculated from oral glucose tolerance test
OGTT	Fasting 2.0-18.4 ulU/ml; 30 min 6.0–86.0 ulU/ml; 60 min 8.0-112.0, 90 min 5.0–68.0 ulU/ml	Marker of body’s insulin response after a glucose load
HDL	> 50 mg/dL	Cholesterol traditionally accepted to be beneficial for cardiovascular health
LDL	< 100 mg/dL	Cholesterol associated with increased cardiovascular disease risk
Triglycerides	< 100 mg/dL	Circulating lipid that may increase related to excess glucose in diet

HDL: high-density lipoprotein, HOMA: homeostasis model assessment, LDL: low-density lipoprotein, OGTT: oral glucose tolerance test.

Because of drastic changes to body composition that accompany SCI due to sarcopenia and neurogenic obesity, standard body mass index (BMI) cutoffs are not accurate in assessing obesity. Specifically, BMI (a standard surrogate measure of body fat percentage using height and weight) underestimates percentage fat mass in patients with SCI^30^. A more appropriate cutoff, given the associated loss of muscle mass, has been proposed to be 22 kg/m^16,19^. Thus, an additional subgroup analysis dividing patients with obesity (BMI *≥* 22) and those without obesity (BMI < 22) were analyzed across body composition, metabolic, blood, and serum variables. A second subgroup analysis determined those with paraplegia defined as those with injury below thoracic level 1 (T1) versus those with tetraplegia defined as those with injuries at and above T1.

A subset of 38 individuals underwent OGTT among whom 27 had insulin resistance assessed by HOMA-IR and the Matsuda Index.

### Statistical analysis

Continuous variables were summarized with median and interquartile range and compared with Wilcoxon Rank Sum test. Categorical variables were summarized with percentage and compared with Fisher’s Exact test. Pearson correlation was used to assess the correlation between different parameters. The significance level for outcomes comparisons was set to the Bonferroni adjusted value of 0.0004 (0.05/116 outcome comparisons). All tests were 2-sided. Statistical analyses were performed in SAS 9.4 (SAS Institute, Cary, North Carolina, United States).

## Results

### Participant demographics and clinical characteristics

A total of 62 individuals (69% male, *n* = 43; 31% female, *n* = 19) with chronic traumatic SCI, mean injury duration 7.4 *±* 5.8 years, were included in the study, Table 2. The mean age of participants was 34.4 *±* 12.1 years. Cervical level of injury was observed in 79% of cases and thoracic in 18%. Severity of injury included ASIA A grade in 61% (*n* = 49), ASIA B 34% (*n* = 21), and ASIA C in 2% (*n* = 1). Approximately 64% had BMI < 25 and 11% > 30.

**Table 2:** Demographics across BMI and injury level cohorts.

			BMI			Level of injury		
		All (n=26)	< 22 (n=13)	*≥* 22(n=13)	p-value	T1 or above (n=22	below T1 (n=5)	p-value
Sex	Female, %	18.52%	30.77%	7.69%		18.18%	20%	
	Male, %	81.48%	69.23%	92.31%	0.3217	81.82%	80%	1
Age (yrs), Median [Q1, Q3]		32 [25, 43]	30 [23, 41]	39 [27, 43]	0.5546	31 [25, 42]	39 [39, 46]	0.2349
BMI, Median [Q1, Q3]		22.65 [18.56, 26.72]	18.56 [18.37, 21.09]	26.72 [25.15, 28.98]	<.0001	22.45 [18.56, 26.25]	22.85 [21.71, 28.37]	0.5365
Time since injury (years), Median [Q1, Q3]		6.66 [3.7, 9.26]	6.64 [3.12, 9.85]	6.96 [4.03, 7.53]	0.9795	6.54 [3.7, 7.3]	9.85 [8.09, 10.16]	0.1341
Level of injury	Cervical, %	77.78%	76.92%	76.92%		95.45%	0%	
	Thoracic, %	22.22%	23.08%	23.08%	1	4.55%	100%	<.0001
AIS score	A, %	48.15%	53.85%	46.15%		50%	40%	
	B, %	48.15%	46.15%	46.15%	1	50%	40%	0.2171
	C, %	3.70%	0%	7.69%		0%	20%	

### Body composition

Mean BMI was 23.8 ± 5, Table 3. Several measures of body fat were significantly correlated with cardiometabolic risk factors. Total and percent truncal fat correlated positively with serum triglycerides, non-high-density lipoprotein (HDL) cholesterol, hsCRP, glucose at 30, 60, and 120 min during OGTT, and HOMA IR. Total and percent truncal fat correlated negatively with HDL cholesterol and Matsuda Index.

**Table 3:** Body composition.

	All (*n* = 26)	BMI			Level of injury		
		< 22 (*n* = 13)	*≥* 22 (*n* = 13)	*p*-value	T1 or above (*n* = 22)	below T1 (*n* = 5)	*p*-value
Total-%Fat, Median [Q1, Q3]	38.5 [30.7, 43]	33.3 [23.1, 40.3]	42.9 [34.9, 47.2]	0.0048	40.6 [33.3, 43]	33.7 [30.7, 34.8]	0.3491
Total-mass (kg), Median [Q1, Q3]	75.7 [60.4, 89]	60.4 [51.5, 72.8]	89 [79.9, 99.4]	0.0002	71.25 [60.4, 89]	79.9 [79.6, 81.2]	0.5325
Total-fat (g), Median [Q1, Q3]	26,559 [16691, 37055]	16,691 [15068, 20253]	37,055 [33305, 41238]	< 0.0001	25838.5 [17941, 37055]	26,559 [16578, 36218]	0.8028
Total-lean (g), Median [Q1, Q3]	47,210 [35825, 54694]	36,369 [34568, 47518]	50,804 [44487, 54694]	0.0687	46258.5 [35825, 52798]	49,655 [40576, 61195]	0.3179
Trunk-%Fat, Median [Q1, Q3]	37.9 [27.3, 46.7]	30.1 [20.1, 38.4]	46 [36.3, 50.3]	0.0032	39.1 [30.1, 46.7]	34.6 [24.7, 36]	0.3491
Trunk-mass (kg), Median [Q1, Q3]	37.7 [30.71, 47.38]	30.71 [25.8, 36.8]	47.38 [42.4, 50.4]	0.0002	36.7 [30.71, 47.38]	38.4 [37.7, 43.5]	0.5325
Trunk-fat (g), Median [Q1, Q3]	12,831 [7722, 19688]	7722 [6217, 9958]	19,688 [18127, 25657]	0.0001	13152.5 [7839, 19688]	12,831 [6781, 19657]	0.9007
Trunk-lean (g), Median [Q1, Q3]	23,204 [19200, 26367]	19,338 [18171, 23204]	24,224 [23760, 26367]	0.0483	23032.5 [18496, 25118]	24,224 [20811, 29915]	0.2612

### Metabolic profile

Mean hemoglobin A1C (HbA1c) was 5.0 *±* 0.6 with 95% of individuals with HbA1c < 5.7, Table 4. Mean HDL was 42.4 *±* 12.7 mg/dL and triglycerides were 113.1 *±* 71.3 mg/dL. Low HDL (< 40 mg/dL) was noted in 47% and high TG in 22% of individuals, Supplementary Table 1. High fasting glucose was noted in 15% and high 2 h glucose in 32% of individuals, Table 5. HOMA IR was > 2.0 in 37% and Matsuda Index < 4.3 in 59% of individuals.

**Table 4:** Serum inflammatory markers and metabolic panel.

		**All (n=26)**	**BMI**				**Level of injury**							
			**< 22 (n=13)**	> 22 (n=13)		**p-value**	**T1 or above (n=22)**			**below T1 (n=5)**		**p-value**		
V-Plex (10-Plex)	IFN-g, Median [Q1, Q3]	1.97 [0.83, 3.39]	2.01 [1.01, 3.65]	1.72 [0.83, 2.41]		0.409	1.97 [0.83, 2.5]			4.34 [1.66, 7.01]		0.3953		
	IL-10, Median [Q1, Q3]	0.27 [0.2, 0.4]	0.29 [0.25, 0.44]	0.22 [0.18, 0.27]		0.1858	0.26 [0.2, 0.44]			0.28 [0.27, 0.29]		0.5254		
	IL-12p70, Median [Q1, Q3]	0.03 [0.02, 0.03]	0.03 [0.02, 0.05]	0.03 [0.03, 0.03]		0.5608	0.03 [0.02, 0.05]			0.03 [0.03, 0.03]		0.4647		
	IL-13, Median [Q1, Q3]	0.68 [0.46, 1.03]	0.75 [0.5, 0.95]	0.6 [0.32, 1.21]		0.8738	0.68 [0.42, 0.95]			0.87 [0.53, 1.21]		0.5254		
	IL-1b, Median [Q1, Q3]	0.03 [0.02, 0.08]	0.15 [0.08, 0.22]	0.02 [0, 0.03]		0.0833	0.03 [0.02, 0.22]			0.04 [0, 0.08]		0.5637		
	IL-4, Median [Q1, Q3]	0.02 [0.01, 0.03]	0.02 [0.01, 0.02]	0.02 [0.01, 0.05]		0.5604	0.02 [0.01, 0.03]			0.02 [0, 0.03]		0.6338		
	IL-6, Median [Q1, Q3]	0.59 [0.3, 0.91]	0.52 [0.27, 0.82]	0.67 [0.5, 1.35]		0.2235	0.53 [0.29, 0.82]			1.29 [0.88, 1.7]		0.1123		
	IL-8, Median [Q1, Q3]	12.79 [8.23, 18.18]	12.78 [7.68, 19.33]	12.79 [8.77, 15.1]		0.8738	12.09 [7.68, 17.03]			24.54 [12.78, 36.3]		0.2664		
	TNF-a, Median [Q1, Q3]	2.54 [1.79, 3.12]	2.52 [1.89, 3.07]	2.56 [1.74, 3.17]		0.9578	2.69 [1.74, 3.17]			2.47 [2.38, 2.56]		0.8738		
U-Plex (6-Plex)	IL-17 A, Median [Q1, Q3]	0 [0, 0]	0 [0, 0]	0 [0, 0]		0.3683	0 [0, 0]			0 [0, 0]		0.153		
	IP-10, Median [Q1, Q3]	40.66 [33.45, 64.85]	57.6 [34.57, 70.7]	36.57 [30.11, 47.6]		0.2664	40.66 [32.32, 59]			171.68 [36.57, 306.79]		0.3408		
	MCP-1, Median [Q1, Q3]	124.39 [98.91, 177.09]	119.4 [69.76, 151.44]	174.33 [117.96, 183.3]		0.1248	120.68 [96.2, 179.85]			162.89 [151.44, 174.33]		0.4273		
	MIP-1a, Median [Q1, Q3]	35.78 [27.99, 44]	33.89 [28.2, 50.68]	37.66 [27.78, 42.64]		0.9578	36.43 [27.78, 45.35]			32.93 [28.2, 37.66]		0.6338		
	MIP-1b, Median [Q1, Q3]	83.48 [55.4, 116.33]	83.05 [59.09, 106.58]	83.92 [54.56, 122.45]		0.8738	83.48 [56.23, 110.22]			84.49 [33.42, 135.57]		0.8738		
	MIP-3a, Median [Q1, Q3]	4.75 [2.72, 6.87]	5.07 [3.33, 6.87]	3.53 [2.62, 6.87]		0.6718	3.98 [2.62, 6.87]			6.4 [5.92, 6.87]		0.3822		
U-Plex (4-Plex)	IL-21, Median [Q1, Q3]	2.57 [1.72, 3.86]	1.8 [1.71, 3.41]	2.76 [1.73, 4.27]		0.5244	2.57 [1.73, 4.27]			2.58 [1.71, 3.46]		0.8736		
	IL-22, Median [Q1, Q3]	1.66 [0.96, 2.48]	1.78 [1.08, 2.89]	1.56 [0.72, 1.84]		0.1858	1.66 [0.87, 2.16]			1.97 [1.05, 2.89]		0.7508		
	IL-27, Median [Q1, Q3]	262.97 [172.07, 375.94]	255.97 [180.36, 440.21]	277.83 [163.78, 359.18]		0.7913	241.71 [163.78, 344.75]			403.28 [359.18, 447.37]		0.1123		
	IL-33, Median [Q1, Q3]	0.55 [0.43, 0.95]	0.57 [0.51, 1.03]	0.48 [0.35, 0.86]		0.2888	0.57 [0.43, 0.95]			0.54 [0.52, 0.55]		0.8651		
hsCRP (mg/L), Median [Q1, Q3]		2.3 [1, 7.9]	1.8 [1, 7.9]	2.3 [1.8, 5.6]		0.6812	2.3 [1.5, 7.9]			2.3 [1, 3.7]		0.8269		
BMP-glucose (mg/dL), Median [Q1, Q3]		91 [87, 96]	88 [86, 93]	93 [91, 96]		0.0642	91.5 [88, 96]			88 [87, 91]		0.1786		
BMP-urea nitrogen (mg/dL), Median [Q1, Q3]		13 [11, 14]	13 [11, 14]	11 [11, 14]		0.8147	11 [10, 13]			14 [13, 16]		0.1127		
BMP-creatinine (mg/dL), Median [Q1, Q3]		0.65 [0.55, 0.73]	0.62 [0.55, 0.67]	0.68 [0.56, 0.73]		0.3831	0.64 [0.52, 0.73]			0.69 [0.56, 0.69]		0.7079		
BMP-eGFR non-aa (mL/min/1.73m2), Median [Q1, Q3]		126 [116, 134]	128 [123, 134]	121 [116, 130]		0.6441	127.5 [118, 136]			120 [114, 123]		0.1184		
BMP-eGFR aa (mL/min/1.73m2), Median [Q1, Q3]		145 [135, 155]	148 [143, 155]	140 [135, 151]		0.6441	147.5 [137, 157]			140 [132, 143]		0.1259		
BMP-bun/creatinine ratio (calc), Median [Q1, Q3]		18.5 [14, 24.5]	19 [16, 24]	16 [12, 25]		0.4567	17 [13, 22]			28 [25, 31]		0.0666		
BMP-sodium (mmol/L), Median [Q1, Q3]		140 [139, 141]	141 [138, 142]	140 [139, 141]		0.6193	140.5 [139, 141]			140 [139, 141]		0.8738		
BMP-potassium (mmol/L), Median [Q1, Q3]		4.3 [4, 4.4]	4.2 [4.1, 4.4]	4.3 [4, 4.3]		0.4841	4.3 [4.1, 4.4]			4.1 [3.8, 4.4]		0.5492		
BMP-chloride (mmol/L), Median [Q1, Q3]		103 [102, 105]	103 [102, 105]	104 [102, 105]		0.8767	103 [102, 105]			104 [103, 105]		0.8502		
BMP-CO2 (mmol/L), Median [Q1, Q3]		24 [21, 26]	24 [21, 26]	24 [22, 26]		0.9177	23.5 [21, 25]			27 [26, 27]		0.0109		
BMP-calcium (mg/dL), Median [Q1, Q3]		9.3 [9, 9.5]	9.1 [9, 9.6]	9.3 [9, 9.4]		0.8163	9.2 [9, 9.5]			9.4 [9, 9.6]		0.6601		
HFP-protein tot. (g/dL), Median [Q1, Q3]		6.7 [6.4, 7.1]	6.8 [6.2, 7.3]	6.7 [6.6, 6.8]		0.5535	6.7 [6.2, 7]			7 [6.8, 7.3]		0.0446		
HFP-albumin (g/dL), Median [Q1, Q3]		4.2 [3.9, 4.4]	4.2 [3.9, 4.3]	4.3 [3.9, 4.3]		0.9587	4.2 [3.8, 4.3]			4.3 [4.1, 4.5]		0.3133		
HFP-globulin (g/dL), Median [Q1, Q3]		2.6 [2.3, 2.8]	2.6 [2.1, 3.1]	2.6 [2.4, 2.8]		0.699	2.5 [2.2, 2.8]			2.8 [2.8, 2.9]		0.0903		
HFP-albumin/globulin ratio (calc), Median [Q1, Q3]		1.6 [1.4, 1.9]	1.6 [1.4, 1.9]	1.6 [1.4, 1.8]		0.8962	1.65 [1.4, 1.9]			1.5 [1.4, 1.6]		0.3579		
HFP-bilirubin tot. (mg/dL), Median [Q1, Q3]		0.5 [0.4, 0.6]	0.5 [0.4, 0.6]	0.4 [0.3, 0.5]		0.2001	0.4 [0.4, 0.5]			0.8 [0.5, 0.9]		0.0752		
HFP-bilirubin dir. (mg/dL), Median [Q1, Q3]		0.1 [0.1, 0.1]	0.1 [0.1, 0.1]	0.1 [0.1, 0.1]		0.1914	0.1 [0.1, 0.1]			0.15 [0.1, 0.2]		0.0338		
HFP-bilirubin indir. (mg/dL), Median [Q1, Q3]		0.4 [0.3, 0.5]	0.4 [0.3, 0.5]	0.4 [0.3, 0.4]		0.9524	0.4 [0.3, 0.4]			0.7 [0.55, 0.8]		0.0108		
HFP-alkaline phosphatase (U/L), Median [Q1, Q3]		70 [59, 83]	63 [59, 72]	74 [60, 83]		0.4114	69.5 [59, 79]			79 [60, 83]		0.4167		
HFP-ast (U/L), Median [Q1, Q3]		17 [14, 25]	20 [15, 27]	17 [14, 25]		0.425	17 [14, 25]			21 [17, 25]		0.5105		
HFP-alt (U/L), Median [Q1, Q3]		18 [12, 26]	19 [11, 25]	18 [13, 26]		0.8373	17 [12, 26]			20 [17, 25]		0.6847		
HgA1C (%), Median [Q1, Q3]		4.9 [4.7, 5.2]	4.85 [4.65, 5.05]	5.1 [4.8, 5.2]		0.216	4.8 [4.7, 5.1]			5.2 [4.9, 5.4]		0.0451		
CBC-white blood cell count (thousand/uL), Median [Q1, Q3]		5.5 [4.9, 7.1]	5.2 [4.6, 5.6]	6.6 [5.5, 7.1]		0.0541	5.55 [4.9, 7.1]			5 [4.9, 6.6]		0.8026		
CBC-red blood cell count (million/uL), Median [Q1, Q3]		4.76 [4.41, 4.96]	4.64 [4.21, 4.9]	4.79 [4.6, 4.96]		0.4886	4.7 [4.41, 4.9]			4.96 [4.67, 5.08]		0.248		
CBC-hemoglobin (g/dL), Median [Q1, Q3]		13.9 [13, 14.5]	13.2 [13, 14.4]	14.1 [13.1, 14.6]		0.3419	13.6 [13, 14.4]			14.1 [13.2, 15.1]		0.4345		
CBC-hematocrit (%), Median [Q1, Q3]		40.8 [38.7, 43.5]	39.8 [38.6, 42.9]	41.3 [39.1, 43.7]		0.4415	40.65 [38.6, 43.2]			41.3 [40.3, 47.1]		0.2005		
CBC-mcv (fL), Median [Q1, Q3]		88.4 [84, 91]	87.5 [83.3, 90]	88.4 [85.4, 89]		0.9591	88.3 [85, 90]			88.4 [81.7, 92.8]		0.8759		
CBC-mch (pg), Median [Q1, Q3]		29.7 [28.1, 30.7]	29.7 [27.5, 30.2]	29.6 [29.2, 30.7]		0.6259	29.65 [28.8, 30.7]			30.2 [26.6, 30.2]		0.662		
CBC-mchc (g/dL), Median [Q1, Q3]		33.6 [33, 34.3]	33.6 [33, 34.1]	33.7 [33.4, 34.4]		0.4721	33.8 [33.2, 34.4]			32.8 [32.1, 33.4]		0.0607		
CBC-rdw (%), Median [Q1, Q3]		13.6 [12.8, 13.9]	13.7 [13.2, 13.9]	13.2 [12.7, 13.7]		0.2802	13.25 [12.7, 13.8]			13.7 [13.7, 14.1]		0.247		
CBC-platelet count (thousand/uL), Median [Q1, Q3]		219 [171, 247]	206 [168, 262]	222 [201, 233]		0.6628	209 [171, 230]			262 [247, 326]		0.098		
CBC-mpv (fL), Median [Q1, Q3]		10.4 [9.6, 11.1]	10.75 [9.4, 11.2]	10.3 [9.7, 11.1]		0.5677	10.75 [9.7, 11.2]			9.75 [8.9, 10.3]		0.0897		
CBC-abs. neutrophils (cells/uL), Median [Q1, Q3]		1313.1 [3.3, 3614]	3.6 [3.1, 3218]	1567.6 [3.4, 3802]		0.5136	4.95 [3.3, 3614]			3101 [1537.3, 3664]		0.6696		
CBC-abs. lymphocytes (cells/uL), Median [Q1, Q3]		557.4 [1.4, 1440]	1.6 [1.3, 1240]	697.4 [1.45, 1975]		0.1416	2.7 [1.3, 1415]			1276 [556.7, 1667]		0.434		
CBC-abs. monocytes (cells/uL), Median [Q1, Q3]		136.8 [0.4, 426]	0.5 [0.3, 473]	142.3 [0.45, 384]		0.9781	0.6 [0.3, 470]			355 [170.2, 398]		0.6164		
CBC-abs. eosinophils (cells/uL), Median [Q1, Q3]		35.15 [0.2, 138]	0.3 [0.1, 158]	35.15 [0.2, 106.5]		0.9343	0.3 [0.1, 138]			74.5 [35.1, 159.5]		0.5184		
CBC-abs. basophils (cells/uL), Median [Q1, Q3]		9.05 [0, 41]	0.1 [0, 39]	10 [0, 39]		0.8422	0.05 [0, 39]			34.5 [10.05, 54]		0.1296		
CBC-neutrophils (%), Median [Q1, Q3]		62.3 [56.5, 67.9]	62.85 [59.25, 69.1]	61.3 [51, 67]		0.2421	61.65 [56.1, 68.9]			62.65 [59.3, 63.15]		1		
CBC-lymphocytes (%), Median [Q1, Q3]		27.2 [20.4, 31.35]	24.5 [20.4, 28.2]	28.8 [17, 33.7]		0.3097	26 [18.4, 31.85]			28.75 [25.7, 29.9]		0.6985		
CBC-monocytes (%), Median [Q1, Q3]		7.65 [5.65, 8.6]	8.6 [6.8, 9]	5.6 [4.9, 8]		0.0049	7.65 [5.65, 8.75]			7.4 [6.2, 8.35]		0.938		
CBC-eosinophils (%), Median [Q1, Q3]		2.65 [1.8, 3]	3 [2.45, 3.05]	1.6 [1.2, 3]		0.0321	2.65 [2, 3]			2.7 [1.3, 4.45]		0.8148		
CBC-basophils (%), Median [Q1, Q3]		0.7 [0.35, 1]	0.8 [0.35, 1]	0.5 [0, 0.9]		0.2851	0.7 [0.15, 1]			0.95 [0.65, 1]		0.2691		
Lipid Panel-Total cholesterol (mg/dL), Median [Q1, Q3]		148 [135, 180]	139 [131, 149]	161 [148, 181]		0.0726	144 [132, 179]			152 [149, 180]		0.2611		
Lipid Panel-HDL Cholesterol (mg/dL), Median [Q1, Q3]		36 [32, 49]	45 [35, 55]	34 [29, 36]		0.0056	35.5 [31, 45]			48 [41, 49]		0.0801		
Lipid Panel-Triglycerides (mg/dL), Median [Q1, Q3]		86 [65, 120]	65 [43, 79]	107 [87, 205]		0.0011	86.5 [66, 121]			84 [43, 103]		0.3654		
Lipid Panel-LDL Cholesterole (mg/dL), Median [Q1, Q3]		87 [70, 112]	82 [70, 91]	112 [85, 127]		0.0311	85 [70, 112]			91 [87, 94]		0.6846		
Lipid Panel-CHOL/HDLC ratio, Median [Q1, Q3]		4.2 [3, 5]	4.2 [3, 4.2]	4.2 [3.7, 6.2]		0.461	4.2 [4, 5.2]			3.35 [2.95, 4.35]		0.3512		
Lipid Panel-NON HDL Cholesterol (mg/dL), Median [Q1, Q3]		52 [13, 106]	13 [11, 100]	89 [24, 115]		0.0255	30 [13, 103]			100 [89, 111]		0.399		
Pre-albumin, Median [Q1, Q3]		24 [21, 28]	23 [21, 27]	24.5 [19, 28.5]		0.6824	24 [21, 28]			23 [18.5, 26.5]		0.5447		
Vitamin D (ng/mL), Median [Q1, Q3]		35.4 [25, 45]	38 [31, 46.5]	27.9 [14.8, 41.5]		0.0576	33.7 [25, 42]			35.4 [29, 45]		0.9254		

**Table 5:** Measure of insulin sensitivity.

		All (*n* = 26)				BMI									Level of injury													
						< 22 (*n* = 13)				*≥* 22 (*n* = 13)			*p*-value		T1 or above (*n* = 22)				below T1 (*n* = 5)	*p*-value								
Glucose-baseline (mg/dL), Median [Q1, Q3]		91 [87, 97]				89 [86, 98]				92 [91, 95]			0.2375		91 [87, 97]				92 [82, 94]	0.5737								
Glucose-30 min (mg/dL), Median [Q1, Q3]		157 [136, 171]				136 [112, 169]				158 [154, 171]			0.1509		158 [136, 171]				146 [110, 158]	0.4168								
Glucose-60 min (mg/dL), Median [Q1, Q3]		159 [130, 173]				142 [109, 164]				165 [153, 181]			0.0647		164.5 [132, 176]				142 [113, 153]	0.0803								
Glucose-120 min (mg/dL), Median [Q1, Q3]		127 [101, 144]				116 [101, 138]				127 [111, 144]			0.7195		129 [110, 145]				111 [101, 138]	0.417								
Insuline-baseline (), Median [Q1, Q3]		7.3 [4.8, 10]				5.1 [3.6, 7.3]				8.5 [6.5, 12.7]			0.0096		7.45 [4.8, 9]				5.8 [5.8, 10]	0.9006								
Insulin-30 min (), Median [Q1, Q3]		79.5 [34, 103.2]				41.5 [30.7, 85.8]				89.8 [70.7, 155]			0.0333		79.9 [34.2, 103.2]				70.7 [30.3, 85.8]	0.5743								
Insulin-60 min (), Median [Q1, Q3]		77 [39.7, 131.1]				49.9 [33, 92.4]				94.1 [62.7, 148.2]			0.1062		88.95 [49.9, 131.1]				32.7 [31, 46.5]	0.0805								
Insulin-120 min (), Median [Q1, Q3]		45.2 [21.6, 123.1]				35.3 [24.7, 58.2]				94.8 [21.6, 148.3]			0.1742		45.55 [24.7, 125.2]				28.2 [21.6, 61.4]	0.4923								
c-peptide-baseline (ulU/mL), Median [Q1, Q3]		1.6 [1.08, 2.2]				1.1 [0.94, 1.67]				1.93 [1.63, 2.61]			0.009		1.71 [1.1, 2.39]				1.3 [0.98, 1.63]	0.227								
c-peptide-30 min (ng/mL), Median [Q1, Q3]		5.8 [4.16, 7.25]				4.87 [3.85, 6.23]				6.29 [4.59, 8.46]			0.2534		6.16 [4.16, 7.51]				4.98 [4.18, 5.8]	0.3198								
c-peptide-60 min (ng/mL), Median [Q1, Q3]		7.97 [5.54, 10.33]				6.56 [5.42, 9.37]				8.17 [5.79, 10.51]			0.6245		8.4 [6.04, 10.68]				6.08 [4.25, 7.25]	0.1552								
c-peptide-120 min (ng/mL), Median [Q1, Q3]		7.38 [4.91, 11.09]				6.94 [5.02, 10.55]				8.14 [4.3, 14.02]			0.6245		7.38 [4.91, 11.09]				6.81 [4.93, 10.19]	0.9433								
Matsuda Index, Median [Q1, Q3]		3.63 [2.73, 8.07]				6.92 [3.75, 8.75]				2.98 [2.41, 3.63]			0.0138		3.63 [2.52, 7.89]				6.62 [3.29, 8.07]	0.6175								
Insulinogenic Index, Median [Q1, Q3]		1.27 [0.73, 1.85]				0.75 [0.54, 1.42]				1.34 [1.07, 2.03]			0.1661		1.23 [0.73, 1.6]				2.03 [0.9, 6.12]	0.3821								
Disposition Index, Median [Q1, Q3]		4.29 [3.12, 7.32]				4.9 [3.15, 9.96]				4.29 [2.98, 5.55]			0.5554		4.29 [3.15, 7.31]				5.55 [3.12, 48.21]	0.4538								
HOMA IR (Methews 1985), Median [Q1, Q3]		1.58 [1.05, 2.26]				1.09 [0.7, 1.58]				2.02 [1.52, 2.94]			0.0111		1.6 [1.05, 2.26]				1.41 [1.31, 2.02]	0.8515								
Glucose, Median [Q1, Q3]		91 [87, 97]				89 [86, 98]				92 [91, 95]			0.2375		91 [87, 97]				92 [82, 94]	0.5737								
Insulin, Median [Q1, Q3]		7.3 [4.8, 10]				5.1 [3.6, 7.3]				8.5 [6.5, 12.7]			0.0096		7.45 [4.8, 9]				5.8 [5.8, 10]	0.9006								
HOMA2%B, Median [Q1, Q3]		77.4 [70.9, 103.2]				75.5 [65.1, 91.6]				95.7 [76.3, 122.8]			0.0378		80.1 [70.9, 97.2]				76.5 [75.4, 122.8]	0.755								
HOMA2%S, Median [Q1, Q3]		106 [79.3, 160]				151.6 [106, 220.6]				86.7 [60.5, 116.6]			0.0083		104.25 [84.8, 160]				129.1 [79.3, 131.5]	1								
HOMA2 IR, Median [Q1, Q3]		0.94 [0.62, 1.26]				0.66 [0.45, 0.94]				1.15 [0.86, 1.65]			0.0089		0.96 [0.62, 1.18]				0.77 [0.76, 1.26]	1								
Neurogenic Bowel Score, Median Q1, Q3]		13 [8, 18.5]				11.5 [6.5, 1.7]				13 [9, 21]			0.4581		13 [6.5, 18.5]				14 [11, 18]	0.5338								
Calories (kcal), Median [Q1, Q3]		1454 [1155, 1629.6]				1565.33 [1105.4, 1734.2]				1377.2 [1155, 1480]			0.2265		1395 [1155, 1629.6]				1548.8 [1292.7, 1999.3]	0.4202								
Carbs (g), Median [Q1, Q3]		139.2 [114, 149]				143.17 [115.4, 208]				126.2 [91, 139.2]			0.0819		139.2 [111, 149]				143.1 [126.9, 197.3]	0.3701								
Fat (g), Median [Q1, Q3]		56.4 [43.6, 69.4]				58.75 [44, 70.2]				48.5 [32, 69.4]			0.2897		51 [42.4, 63.2]				62.9 [50, 89.5]	0.3703								
Protein (g), Median [Q1, Q3]		62 [49, 78]				58.6 [49, 82]				57 [46, 71.4]			0.6229		52 [48, 74.2]				84.1 [59.5, 107.1]	0.1067								
Cholest (mg), Median [Q1, Q3]		169 [114.8, 254.6]				198.7 [133.4, 255]				154.7 [99, 201]			0.3643		140.4 [102.25, 255]				180.9 [171.7, 221]	0.4737								
Sodium (mg), Median [Q1, Q3]		2223 [1678, 2844.2]				2273 [2117.2, 2980]				1653.5 [1494, 2459]			0.0696		2187.2 [1629, 2459]				3031.7 [2480.7, 3777.6]	0.0732								
Sugars (g), Median [Q1, Q3]		43.4 [32, 58]				42.7 [35, 49.6]				38 [31, 58]			0.7623		42[32, 58]				46.5 [34.7, 59]	0.8578								
Fiber (g), Median [Q1, Q3]		10 [6.25, 15]				11.6 [8, 17]				9 [6, 15]			0.1854		9.2 [6, 14]				14.5 [10.8, 17.8]	0.1066								

### Inflammatory profile

Inflammatory marker CRP correlated positively with BMI (*r* = 0.57), total fat (*r* = 0.42) and truncal fat (*r* = 0.43), see Supplementary Table 1. Additionally, IL-8 correlated positively with HbA1c (*r* = 0.69), serum glucose (*r* = 0.62), BMI (*r* = 0.34), triglycerides (*r* = 0.43), total lean mass (*r* = 0.42) and truncal lean mass (*r* = 0.38). Interleukin 27 correlated with neurogenic bowel score (*r* = 0.42).

### BMI and level of injury subgroup analyses

Patients were dichotomized into below BMI 22 (*n* = 13) and BMI *≥* 22 (*n* = 13). Of those with BMI below 22, 31% were female with median age of 30 years. Of those greater than or equal to 22, 8% were female with median age of 39 years. The population was also dichotomized into those with paraplegia (below T1 injury level) (*n* = 5) versus tetraplegics (at and above T1 injury level) (*n* = 22). For those with paraplegia, females made up 18% with median age 31, half were ASIA A and the other half ASIA B. Those with tetraplegia were 20% female and median age of 39, 40% ASIA A, 40% ASIA B, 20% ASIA C.

For those with BMI *≥* 22, body fat % was higher when compared to those with BMI < 22 (42.9 vs. 33.3%, *p* = 0.0048), as was total mass, total fat, trunk mass, trunk fat, and trunk lean mass. Body composition metrics were not statistically different between injury level groups, for those with paraplegia versus tetraplegia.

Baseline insulin and c-peptide were higher for those with BMI ≥ 22 compared to BMI < 22 (8.5 vs. 5.1 mlU/L, *p* = 0.0096) and 1.93 versus 1.1 mlU/L, *p* = 0.009), as was 30-minute insulin on OGTT (89.8 versus 41.5 mmol/L, *p* = 0.0333). All measures of OGTT were similar for patients above and below T1 injury levels. Matsuda Index was lower for those with BMI *≥* 22 (2.98 versus 6.92, *p* = 0.0138) and HOMA-IR was higher (2.02 versus 1.09, *p* = 0.0111) when compared to those with BMI < 22. HOMA2%B was higher for the BMI *≥* 22 group (95.7 versus 75.5, *p* = 0.0378), lower for HOMA2%S (86.7 versus 151.6, *p* = 0.0083), and greater for HOMA-IR (1.15 versus 0.66, *p* = 0.0089). Matsuda and HOMA values were similar between injury level groups.

Those with BMI *≥* 22 HDL had worse lipid profile values with lower HDL (34 versus 45 mg/dL, *p* = 0.0056), higher triglycerides (107 versus 65 mg/dL, *p* = 0.0011), higher low-density-lipoproteins (LDL) (112 versus 82 mg/dL, *p* = 0.0311), and overall non-HDL cholesterol (89 versus 13 mg/dL, *p* = 0.0255). No differences were observed in lipid profile lab values between injury level groups. Serum values assessing inflammatory profiles and neurogenic bowel score were similar between both BMI and injury level subgroups. All data generated or analyzed during this study are included in this published article [and its supplementary information files].

## Discussion

Before 1970, urogenital complications were a leading cause of mortality in SCI^31^. As urinary and pulmonary infections have been better controlled and greater longevity is seen in those with SCI, cardiovascular disease is now a leading cause of death^5,31^. Due to the ensuing energy imbalance with less physical exercise, adiposity, and higher risk of cardiometabolic complications, cardiovascular disease is a leading cause of mortality in SCI^19^. Those with SCI become physically deconditioned following injury, leading to physiologic alterations and reduction in whole-body metabolism^19^. Cellular, hormonal, tissue, and organ system changes following SCI predispose to greater metabolic risk profiles that lead to premature onset of metabolic syndrome and CMD^8,9^. Traumatic SCI has a higher 5-year accumulation of CMD risk factors compared with able-bodied adults (56% vs. 36%) and more than tripled risk for heart failure^1^. There is an elevation of 47% in mortality for those with SCI over 4.5 years in one study and the most common cause of death was circulatory diseases at over 40%^4^. The present study demonstrates increased risk of CMD in those with chronic SCI in metabolic, body composition domains, particularly for those with BMI > 22. Moreover, the impact of obesity and truncal fat—independent of time since injury and level of injury—appear to have a major role in CMD risk in those with SCI.

Metabolic syndrome has been observed in as many as 55% of those with chronic SCI^32^. Several sources assert that tetraplegia is associated with greater CMD risk and body composition deterioration than is paraplegia^8,15,33^. One study showed that patients with tetraplegia tend to have 5% higher fat mass than those with paraplegia^33^. Other studies have reported no changes in body composition between tetraplegia versus paraplegia or even worse body composition for those with paraplegia^34,35^. The current study shows that body composition, independent of injury level, is associated with elevated CMD risk. Moussavi and colleagues demonstrated that time since injury was more associated with severity of dyslipidemia^36^. However, the present study showed that time since injury was not correlated with body composition changes nor CMD profile risk.

### Decrements in body composition

Truncal or central obesity has been particularly implicated as a driver for cardiometabolic disease risk in those with SCI^19,37^. Central or visceral fat is metabolically active with adipokines increasing systemic inflammation^5,6,37,38^. Additionally, regional fat distribution with higher waist circumference and deposition of visceral fat has been associated with higher risk of dyslipidemia, insulin resistance, and greater than 10% risk of heart attack in 10 years (Framingham risk score) than BMI or other fatty body composition^8,37^. Given energy expenditure differences after injury, those with SCI are at greatest risk of building central adiposity, especially in the first year following injury^19,39^.

In the general population, a ratio of 0.4 subcutaneous adipose tissue (SAT) to visceral adipose tissue (VAT) ratio is associated with higher risk of developing CMD; whereas, those with SCI are reported to have a SAT to VAT ratio of 0.7^5,38^. The present study demonstrates that BMI *≥* 22 kg/m was related to higher risk of CMD with higher truncal fat, worsened lipid profile, greater insulin insensitivity when compared with those without obesity. When the sample was dichotomized by level of injury, no changes were observed in CMD risk. Clinical significance of BMI 22 appears to indicate an appropriate cutoff to demonstrate CMD risk^16^. Our findings are inconsistent with prior literature regarding increased risk of poor body composition and subsequent CMD risk in those with tetraplegia. Spungen et al. demonstrate that those with tetraplegia had greater total and regional obesity than those with paraplegia^34^. Additionally, we showed that time of injury was not associated with worsening body composition and CMD profile, inconsistent with prior literature.

Significant loss in muscle mass from atrophic wasting is associated with both decreased strength and ectopic fatty deposits in skeletal muscle^5,40^. Also intramuscular fat (IMF) or intermuscular adipose tissue (IMAT) deposition and muscle loss perhaps represent a driver for loss of insulin sensitivity, and it increases over time in chronic SCI^41,42^. These changes in muscle composition and whole body composition that result from inactivity may underlie alterations in carbohydrate and lipid metabolism that contribute to fat accumulation and insulin resistance^5,43^. Moreover, visceral adipocytes and adipokines secrete and recruit non-esterified fatty acids that systemically contribute to elevated LDL, lower HDL, and ultimately infiltrate skeletal muscle that may perpetuate insulin resistance^8,44^.

### Greater insulin resistance

Insulin resistance is believed to precede diagnosis of DM, ischemic heart disease, stroke by 10–15 years, representing an insidious form of metabolic change that underlies non-communicable disease of aging and cardiovascular dysfunction^29^. Those with BMI *≥* 22 had Matsuda value near 7 while those with BMI < 22 had value of 2.98. Additionally, HOMA Index estimates hepatic influence of insulin resistance where values greater than 2.0 indicate insulin resistance^45^. Those with BMI *≥* 22 had Matsuda greater than 2.0, whereas those with BMI < 22 did not. Baseline insulin levels and c-peptide were also significantly higher in those with obesity.

Skeletal muscle represents 70% of the reservoir for circulating glucose^46^. Physical exercise is also known to recruit translocation of GLUT4 receptors and increased glucose uptake^47^. Those with SCI have greater risk of developing insulin resistance largely due to significantly decreased skeletal muscle mass (shown to have nearly 50% cross-sectional area of muscle loss below level injury within 6 weeks)^48^. Given this higher risk of insulin resistance following SCI, future studies may evaluate the need for different cutoffs in patients with SCI regarding HOMA or Matsuda.

### Increased inflammation

Inflammation has been implicated in metabolic dysfunction in obesity. Increases in fat mass induce inflammatory biomarkers that interfere with insulin signaling and trigger metabolic syndrome^5,49^. SCI is associated with higher systemic inflammatory profile even in absence of acute infection^39^. CRP is associated with CMD in SCI and has been specifically associated with higher risk of cardiac events^19,50^. CRP was positively correlated with BMI, total, and truncal fat in the present study, consistent with prior literature that obesity relates to chronic inflammatory state. However, IL-6 and CRP were not observed to correlate with time of injury or body composition, or group analysis of BMI or time of injury.

Serum IL-8 and MCP-1 are chemokines that are increased in relation to obesity and diabetes, and known to contribute to insulin resistance, chronic inflammation and metabolic dysfunction^11,51,52^. Exercise regimens have been shown to reduce serum MCP-1 and IL-8 over time^53^. They have both been implicated in atherosclerosis and trigger adhesion to vascular endothelium as potential active roles in atherogenesis^54^. In the correlational analysis, total and truncal fat correlated with HbA1c and glucose OGTT. HbA1C was significantly positively correlated with IL-8. This correlation is consistent with prior literature in the able-bodied population that DM has an association with systemic IL-8 elevation^55^. The role of IL-8 in insulin sensitivity is unclear.

We also demonstrate an increase in MCP-1 with lean muscle mass. IMAT secretes MCP-1 that directly mediates insulin resistance and inflammatory cascades in obesity^56–58^. DEXA has been shown to be less accurate in determining truncal lean muscle mass as it has been proven to be difficult to separate from viscera and fluids in the trunk region^5,59^. Higher abdominal adiposity correlates with higher lean muscle mass in older adults^60^. DEXA overestimates truncal lean mass and may include VAT or trunk central adiposity^61^. Moreover, DEXA has not been able to isolate or differentiate IMAT from lean muscle mass and has been incorrectly calculated in the lean muscle mass estimates^62^. The higher the IMAT, the higher the lean muscle mass estimate. IMAT is thought to be metabolically active, and our study demonstrates the likely candidate adipokines that lead to insulin resistance, all correlating positively with lean muscle mass and therefore IMAT.

MIP-1b is implicated as an adipokine of visceral adipose tissue and correlates with BMI, insulin resistance, and dyslipidemia^63,64^. It is possible that lean muscle mass on DEXA may erroneously include ectopic intermuscular adipose tissue—or intramuscular adipose tissue—that is observed to positively correlate with MCP-1, IL-8, and MIP-1b and therefore contribute to inflammatory pathogenesis of metabolically unhealthy IMAT.

IL-27 has recently been implicated in gut barrier health^65,66^. Constipation and bowel dysfunction may contribute to a pathogenic imbalance in the gut microbiome^67,68^. Bowel dysfunction may increase leaky gut and bacterial translocation from the gut^68^. Neurogenic bowel dysfunction score (NBDS) represents a validated assessment of bowel dysfunction in SCI such that the higher the score, the more dysfunction^67^. Further, it may act as a proxy of gut motility that has been shown to predict the negatively affected quality of life^67^. Positive correlation of IL-27 and NBDS indicates a potential relationship with inflammation. IL-27 has been associated with the role of upholding gut intestinal wall lining integrity and repair and pathogen defense^65^. SCI induces gut dysmotility with impairment of the enteric nervous system prolonging bowel transit time and increasing constipation^8,26^. It is unclear if this correlates with alterations in gut microbiome. Further investigation of the gut microbiome in assessing microbiome profiles in those with SCI may elucidate influence and risk of systemic inflammation and body composition profiles that increase risk of CMD.

### Strengths and limitations

The current work is based on a single-institution cross-sectional study on chronic SCI, evaluating multiple aspects of physiological function. Small sample size, particularly in those with paraplegia is a limitation of the subgroup analysis related to injury level. Another limitation of the present study is its correlational arm design, which decreases generalizability. Demographic homogeneity also limits generalizability of findings. A prospective study including an able-bodied control group may enhance significance of findings and delineate differences between patients with SCI and able-bodied matches. To identify key variables that initiate or play larger or initial roles in metabolic dysregulation, future studies may implement randomized groups to evaluate the effects of diet and exercise independently. Additionally, lack of detailed information regarding patient sleep, pain, medication use, and diet are limitations of the present study that may have influenced measured variables on body composition and inflammation. Interventions to improve body composition with efforts related to nutrition, sleep, pharmacy, and rehabilitation that improve voluntary movement with technological aids including spinal cord epidural stimulation may improve metabolic profiles and stave off CMD risk in this particularly vulnerable population^27,69,70^.

## Conclusion

Following SCI, total and truncal fat appear to be associated with cardiometabolic risk factors, including dyslipidemia, obesity, and elevated inflammatory markers, including IL-8 and CRP. BMI *≥* 22, more than level of injury or time since injury, is linked to greater central obesity/VAT (% truncal fat, trunk mass), dyslipidemia (CMD risk factor), insulin resistance and sensitivity and other CMD risk factors. Level of injury does not appear to drive inflammation in peripheral blood or gut dysfunction. Given our findings, the impact of nutrition, exercise, sleep, and neuromodulation technologies targeting body composition changes could be explored to improve CMD risk profiles.

### Transparency, rigor and reproducibility statement

The institutional review board (IRB) approved the present study with IRB# 16.0179. All participants were medically approved for enrollment in an outpatient, standardized program at the University of Louisville, Frazier Rehab Institute. Sixty-two individuals with chronic spinal cord injury were recruited and the following metrics were analyzed: body composition measured by DEXA scan, metabolic profile, lipids, hsCRP, a battery of inflammatory cytokines through serum sample. Dichotomized groups separated by BMI *≥* 22 and those with BMI < 22 as well as those with injury level above T1 and below T1 were analyzed. Continuous variables were summarized with median and interquartile range and compared with Wilcoxon Rank Sum test. Categorical variables were summarized with percentage and compared with Fisher’s Exact test. The significance level for outcomes comparisons was set to the Bonferroni adjusted value of 0.0004 (0.05/116 outcome comparisons). All tests were 2-sided. Statistical analyses were performed in SAS 9.4. All results are available upon request from readers.

## Supplementary Information

Below is the link to the electronic supplementary material.

Supplementary Material 1

## Data Availability

All data and statistics are available upon request. Correlational analyses are available as supplementary data in the manuscript submission. Data can be requested from Nicholas Dietz, nkd25@georgetown.edu.
